# Editorial: Phenotypic characterization, genetics and genomics of livestock in low input systems

**DOI:** 10.3389/fgene.2022.1013177

**Published:** 2022-09-08

**Authors:** Mohammed Ali Al Abri

**Affiliations:** Department of Animal and Veterinary Sciences, College of Agriculture and Marine Sciences, Muscat, Oman

**Keywords:** genetics, genomics, low input production system, livestock, phenotypic characterization

This Research Topic includes a collection of research papers that highlighted various genetic aspects of indigenous breeds in different countries around the globe. The topic discussed the recent advances, current challenges and latest developments in phenotypic characterization and genetics and genomics of livestock in low input production systems. The research conducted varied from Genome Wide Association Studies (GWAS) and selection signatures to estimation of genetic parameters and heritability to genetic background and genetic diversity of indigenous breeds.

The first manuscript by Sutera et al., implemented a GWAS based on DEBV to identify genomic regions putatively associated with mastitis using somatic cells count (SCC) as a proxy. Their research, which was carried out in local Valle del Belice sheep, highlighted eight genomic markers associated with somatic cell score that harboured candidate genes related to udder conformation and the immune system. These finding could prove very useful for the fate of this and other sheep breeds in the Mediterranean basin countries as the dairy sheep production there usually relies on well adapted local breeds. Another manuscript implementing GWAS was by Mancin et al. who implemented a single step GWAS to analyse Beef Traits in Local Alpine Breed. Their study revealed the diversity of the pathways and some novel genes impacting a number of beef traits. The discovery of these new pathways and genes shows the importance of genetic research in local breeds which can expand to global livestock populations. Habimana et al. conducted a GWAS to study of growth performance and immune response to Newcastle disease virus of indigenous chicken in Rwanda. Their results showed several genes implicated in body weight and New Castle disease antibody variation.

An alternative approach to gene discovery is using a candidate gene approach in which a number of selected genes are selected and genotyped in a population of interest. Verma et al. implemented this approach to discover a panel of 10 reference genes in Indian cattle populations adapted to hot arid normoxia and cold arid hypoxia environments. All together, the previous studies open the door for the discovery of causative genes that could be utilized in selection programs in these local breeds in future.

Searching for Selection Signatures is another method for discovery of genes impacting traits of interest. Unlike GWAS, it relies on discovery of regions at which nucleotide variations is reduced due to the rise of a new beneficial mutation. The research article by Serranito et al. was on searching for Selection Signatures Related to Trypanosomosis Tolerance in African Goats. Trypanosomosis is a parasite that greatly impacts various livestock species including camels, cattle, sheep and goats with very little research focusing on it in goats. Therefore, examining selection signatures of innate immunity to it is of vital importance to goat production in Africa. The researchers found 33 selection signatures, 18 of which overlapped previously published research. Such findings are crucial for goat production as they could eventually lead to discovery of genes/allelic combinations of major role on immunity to trypanosome infections in goats. Another attempt to search for selection signatures and genes responsible for trypanotolerence was made by (Yougbaré et al.). In this article, the researchers reported several genes potentially involved in tolerance to trypansoma resistance in the Baoulé x Zebu crossbred cattle in Burkina Faso. In addition to the two previous articles, the selection signatures in West African indigenous cattle of Benin was investigated by (Vanvanhossou et al.). In this study, the results unraveled some genes related to production/economic traits in hybrid Beninese cattle and genes related to immunity and feed efficiency in local Somba cattle.

A key element of this article collection is research in genetic improvement of local breeds in low input systems. Such improvement is the corner stone of future utilization of these breeds and can help complement their adaptation traits with production traits of interest. The first step to any genetic improvement program is the estimation of genetic parameters of the traits to be improved by the program. Two articles in this article collection discussed genetic parameters estimation in low input systems breeds. The first article discussed the estimation of genetic parameters of type traits in first parity Slovenian Cika cows using a generalized linear model procedure (Simčič et al.). In this article, the authors reported medium to high heritabilities for body frame and autochthonous traits and low to medium heritabilities for scored udder traits. These heritabilities are already in use in the prediction of breeding values in Cika cattle which is exemplary for other breeds in other low input systems. The second article was by Hako Touko et al. in which the authors estimated the heritabilities of antibody response to Newcastle disease vaccination impacting local chicken in Cameron. Due to the lack of a large sample size or pedigree data (common to low input systems), the authors used three alternative methods to estimate heritabilities. The first was the breeder’s equation method, the second method was the graphical method and the third was the full-sib/half-sib method. The authors reported low but similar estimates of antibody response to vaccination and a very low estimate of heritability for survival. Nevertheless, the authors reported a significant increase in antibody response when crossing cocks and hens under 1% and 3% selection intensity respectively.

A number of articles in this collection have explored the genetic and phenotypic background of local breeds including (Alaqeely et al. and Samaraweera et al.). In the former, the authors analysed the sequence variation in the mtDNA control region of Dromedary Camels. Their results showed that small genetic differences exist between camel types. They reported two haplogroups shared between almost all dromedary camels with one of them being more common in African dromedary camels. In addition, in order to better characterize camel populations, the authors advise future researchers to use WGS or SNP data which is the same advise provided by Piro. In the later article, the authors reported high genetic diversity but absence of population structure in local chickens of Sri Lanka using Microsatellite Markers. In contrast to the findings reported in the previous article, this research showed high genetic diversity in Sri Lanka’s local chicken breeds which translates to a large potential for the development of locally adapted genetically improved chicken breeds. On the other hand, Kebede et al. used Species distribution models (SDMs) and Phenotypic distribution models (PDMs) to phenotypically characterize the relationship between environmental variation and phenotypic differentiation in Ethiopian indigenous chicken. Their results identified three major ecotypes that show phenotypic differentiation in addition to nine major environmental variables conducive to habitat suitability. In another study, Ouhrouch et al. used Whole Genome Sequencing (WGS) data on 87 sheep to study the genetic uniqueness Moroccan sheep belonging to the five major sheep breeds. Their study assessed the genetic diversity, effective population size, population structure and relationships between the breeds. In addition, they were able to find interesting intra-breed selection signatures. Their study showed that the Morocan breeds are not highly genetically differentiated but that they harbor high genetic diversity and effective population size which makes them a suitable reservoir for the development of adaptive traits against the threats of climate change. The study by Babigumira et al. aimed at characterizing the genetic ancestry and inbreeding levels in the local a population of smallholder pigs in Uganda using 422 pigs. The authors used porcine GeneSeek Genomic Profiler (GGP) 50 K SNP Chip to infer the genetic ancestry as well as inbreeding levels. The study showed a relatively low inbreeding in the breed and revealed a genetic background of the breed that is a mix of old British and modern pig ancestries. Pig production, which is managed mostly through low input systems, is one of the main sources of income for African small holder producers.

The findings in the previous research articles highlight the importance of preserving local breeds for their notable adaptation to various vector borne diseases. Furthermore, identification of genetic regions/genes can help integrate them from genomes of local lower-production breeds to genomes of other breeds with higher production abilities. Therefore, this article collection will serve as a stepping stone towards the characterization, development and genetic improvement of numerous livestock in low input systems.

